# Economic impact of an increased incidence of invasive mold infections (IMI)

**DOI:** 10.1186/1753-6561-5-S6-P51

**Published:** 2011-06-29

**Authors:** C Van Delden, A Iten, A Gayet-Ageron, V Camus, Y Martin, Y Chalandon, D Pittet

**Affiliations:** 1 Service of Infectious Diseases, HUG, Genève, Switzerland; 2 Infection Control Program, HUG, Genève, Switzerland; 3 Service of Haematology, HUG, Genève, Switzerland

## Introduction / objectives

In July 2010, an increase of IMI was observed in patients with leukemia and bone marrow transplantation admitted to the hematology wards at the University of Geneva Hospitals.

## Methods

We conducted a retrospective survey which identified 6 cases of IMI (3 possible and 3 probable) in 2009 and 18 (9 possible, 7 probable and 2 proven) in 2010. All cases occurred during chemotherapy for acute leukemia, allogeneic hematopoietic stem cell transplantation or graft versus host disease. The incidence of nosocomial IMI among the at risk population was 3.6 fold higher in 2010 than in 2009.

We aimed to identify any potential failures in the care of at-risk patients. Additional preventive measures were set up for isolation rooms and their cleansing, and also their computer equipment. Protective measures were instituted for the patients (masks, gowns, caps, food, objects made available for the patients, transportation). Standard antifungal prophylaxis was changed from fluconazole 100 mg/d to voriconazole 400 mg/d.

## Results

The costs of the various preventive measures to stop the occurrence of new fungal infections were estimated at 2'500'000 CHF (1'900'000 Euros) covering the period from June, 2010 to June, 2011. This represented an average of 22’600 CHF (17’176 Euros) per patient admitted. Of note, 31% of the costs were associated with the change in antifungal prophylaxis.

To date no new cases have been reported since the introduction of these measures.

## Conclusion

A supplementary review is in progress in order to quantify the overall impact of the preventive measures taken and to decide which measures should be continued.

## Disclosure of interest

None declared.

